# Variation in Nicotine Metabolization According to Biological Factors and Type of Nicotine Consumer

**DOI:** 10.3390/healthcare11020179

**Published:** 2023-01-06

**Authors:** Hipólito Pérez-Martín, Cristina Lidón-Moyano, Adrián González-Marrón, Marcela Fu, Raúl Pérez-Ortuño, Montse Ballbè, Juan Carlos Martín-Sánchez, José A. Pascual, Esteve Fernández, Jose M. Martínez-Sánchez

**Affiliations:** 1 Group of Evaluation of Health Determinants and Health Policies, Department of Basic Sciences, Universitat Internacional de Catalunya, Carrer de Josep Trueta s/n, 08195 Barcelona, Spain; 2 Tobacco Control Unit, Cancer Prevention and Control Program, Institut Català d’Oncologia, 08908 Barcelona, Spain; 3 Tobacco Control Research Group, Epidemiology and Public Health Program, Institut d’Investigació Biomèdica de Bellvitge—IDIBELL, 08908 Barcelona, Spain; 4 School of Medicine and Health Sciences, Universitat de Barcelona, 08007 Barcelona, Spain; 5 Center for Biomedical Research in Respiratory Diseases (CIBERES), Instituto de Salud Carlos III, 28220 Madrid, Spain; 6 Group of Integrative Pharmacology and Systems Neuroscience, Neurosciences Programme, IMIM (Hospital del Mar Medical Research Institute), Parc de Recerca Biomèdica de Barcelona, 08003 Barcelona, Spain; 7 Addictions Unit, Institute of Neurosciences, Hospital Clínic de Barcelona, 08036 Barcelona, Spain; 8 Department of Medicine and Life Sciences, Universitat Pompeu Fabra, Barcelona Biomedical Research Park (PRBB), 08003 Barcelona, Spain

**Keywords:** biomarker, cotinine, electronic cigarette, nicotine, nicotine metabolism, smoking

## Abstract

This study aims to describe the nicotine metabolite ratio among tobacco smokers and electronic cigarette (e-cigarette) users and nonusers. We analyzed pooled data from a longitudinal and a cross-sectional study of the adult population from the city of Barcelona. The final sample included information on 166 smokers, 164 e-cigarettes users with nicotine, 41 e-cigarette users without nicotine, 95 dual users (users of both products), and 508 nonusers. We used log-linear models to control for the potential confounding effect of the daily number of cigarettes smoked. Salivary nicotine metabolic rate assessment included the rate of nicotine metabolism (cotinine/nicotine) and the nicotine metabolite ratio (trans-3′-hydroxycotinine/cotinine). Exclusive users of e-cigarette without nicotine have the lowest rate of nicotine metabolism (Geometric mean: 0.08, *p*-values < 0.001) while cigarette smokers have the highest (Geometric mean: 2.08, *p*-values < 0.001). Nonusers have lower nicotine metabolic rate than cigarette smokers (Geometric means: 0.23 vs. 0.18, *p*-value < 0.05). Younger individuals (18–44 years) have a higher rate of nicotine metabolism than older individuals (45–64 years and 65–89) (Geometric means: 0.53 vs. 0.42 and 0.31, respectively, *p*-values < 0.01) and individuals with lower body mass index (21–25 kg/m^2^) have a higher rate of nicotine metabolism than the rest (26–30 kg/m^2^ and 31–60 kg/m^2^) (Geometric means: 0.52 vs. 0.35 and 0.36, respectively-values < 0.01). Nicotine metabolic rates are useful biomarkers when reporting smoking status and biological differences between individuals.

## 1. Introduction

Nicotine is a natural compound found in tobacco leaves. Several tobacco products such as cigarettes, oral snuff, pipe tobacco, cigars and chewing tobacco have approximately 2% of nicotine per unit [1]. The most frequent nicotine concentration in the liquid of electronic cigarettes (e-cigarettes) ranges from 0 to 36 mg/mL in high nicotine vaping products [1]. The harmful health effects of tobacco consumption are well known, as it has been associated with more than 25 types of cancer and cardiovascular diseases, among other conditions [2,3,4].

High-dose nicotine absorption in the body happens mainly through smoking or exposure to tobacco smoke. Once tobacco smoke is inhaled, nicotine is absorbed into the organism. After this, the clearance of the compound results in its conversion into better assimilable metabolites. Nicotine is almost entirely degraded to cotinine and cotinine is mostly degraded to trans-3′-hydroxycotinine; both transformations are catalyzed by the enzyme CYP2A6 [5,6,7]. Both compounds have been well studied due to their relative in vivo long half-life (around 16–20 h and 6 h, respectively) [5,8]. Cotinine and trans-3′-hydroxycotinine can be measured in human fluids such as saliva, plasma, blood or urine during ten days after nicotine metabolization [5,9]. Around 80% of nicotine is metabolized to cotinine, turning cotinine into a well-fitted biomarker of tobacco consumption and exposure [5,9]. However, different biological matrixes represent measurements of the same biomarker taken at different points in the pharmacokinetic pathway, as seen with the higher concentration of cotinine in urine in comparison with that in saliva or plasma [10,11]. In addition, the metabolization of nicotine can be affected by various factors. Some of the most common in the literature are gender, age and BMI. In the case of sex, it has been found that sex hormones play a role in the metabolism of cotinine and that women have a faster nicotine metabolism than men. In the case of age and BMI, the bibliography is not so clear in this regard. This is because there are countless factors that can influence the liver (and consequently the CYP2A6 enzyme), sometimes even in contradictory ways [12,13,14].

To study nicotine addiction and smoking behavior, ratios are a great alternative to work with [15]. One of the most intuitive ratios is the rate of nicotine metabolism (RNM), obtained by dividing the concentration of cotinine by that of nicotine (cotinine/nicotine) [16,17]. Even so, as the half-life of nicotine is so short (2 h) [15], these ratios are heavily reliant on the time since the last cigarette was smoked and the number of cigarettes smoked, so the metabolic ratio of nicotine is highly variable during the day [16,18]. To overcome the problem, the use of the nicotine metabolite ratio (NMR), that is, the ratio of trans-3′-hydroxycotinine to cotinine, is further encouraged [17]. The NMR is consistent in different biological fluids (e.g., a person who is determined to be a slow metabolizer by one method is highly likely to be below the cut point for slow metabolism in other fluids, but the cut points may be different between biological matrixes) and captures both inter-individual and environmental differences. Besides, it is more stable than the RNM, meaning that it has minimal variation during the day, lower dependence on time since the last cigarette in smokers and it is stable over a year time (the NMR continues to give very similar values even a year after a reduction in tobacco consumption has begun. This quality also applies to people who have recently quit smoking) [17,19]. The NMR values in saliva and urine can be used as proxies of the NMR in plasma, although values in urine are more variable [5]. Moreover, the NMR is consistently associated with the activity of the enzyme CYP2A6 [5,6,7].

The NMR has been validated as a reliable biomarker of nicotine metabolism and cigarette dependence, especially in saliva and urine, whose collection uses non-invasive techniques [17,20]. On the other hand, while nicotine metabolites have been extensively studied in smokers, information about nicotine metabolites in users of e-cigarettes is scarce [21]. Some studies analyzing tobacco-specific biomarkers among users of e-cigarettes showed that despite its commercialization as a smoking cessation aid, nicotine and cotinine concentrations may be higher in e-cigarette’ users, making e-cigarettes even more addictive than traditional cigarettes [22,23]. Therefore, to examine the variability in nicotine metabolism, the objective of this study is to investigate potential variations in the RNM and NMR in tobacco smokers and users of e-cigarettes according to different biologic factors and smoking status.

## 2. Materials and Methods

**Figure 1:**
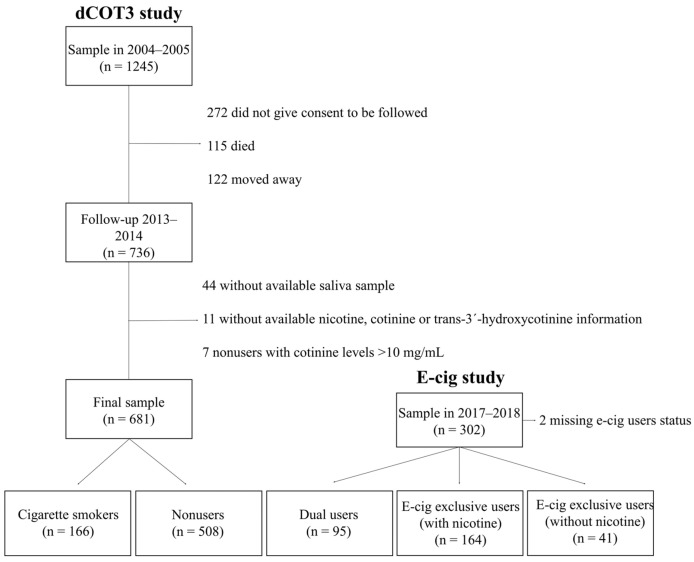
Flow chart of the selection of individuals from the dCOT3 and E-cig studies (*n* = 974).

This is a pooled analysis carried out with data retrieved from two different studies. The first one was a longitudinal study on tobacco smoking patterns “Determinants of Cotinine project-phase 3 (dCOT3 study).” This was a cohort study of a sample of adults (≥16 years at baseline) from the general population of Barcelona (Catalonia, Spain). The baseline was carried out during the years 2004–2005 (*n* = 1245) and one follow-up was carried out during 2013–2014 (*n* = 736). The study included self-reported information about smoking patterns and tobacco exposure, and a saliva sample was collected at the follow up for the determination of various biomarkers of tobacco exposure. After cleansing the follow-up [24], removing subjects that did not have available saliva samples (*n* = 44), subjects without available nicotine, cotinine or trans-3′-hydroxycotinine information (*n* = 11), and seven nonusers whose cotinine concentrations were incompatible with being a nonuser (>10 ng/mL) [25], this sample retained data of 674 individuals. The second study was a cross-sectional study (*n* = 302) conducted in 2017–2018 containing data on adult (≥18 years old) users of e-cigarettes living in Barcelona. As an alternative to a probabilistic sampling technique, the consumer panels technique [26] was used in order to enroll users of e-cigarettes. Although this technique renders the sample unrepresentative of the general population, it minimizes the limitations of the reduced sample size, given the low prevalence of use in this population. Individuals who declared to be current users of e-cigarettes were asked to take part in the study. A questionnaire on e-cigarette use patterns was used and a saliva sample was also collected to determine nicotine, cotinine and trans-3′-hydroxycotinine. Two individuals whose information was missing and could not be categorized were excluded, rendering a second sample of 300 e-cigarette users. Thus, the final merged sample retained data of 974 individuals (Figure 1). The design and methodology of both studies are detailed elsewhere [25,26]. Both studies received approval by the ethics committee of the Bellvitge University Hospital (PR118/11 y PR133/15, respectively) and all participants signed informed consent.

### 2.1. Determination of Biomarkers in Saliva and Computation of the Rate of Nicotine Metabo-Lism and Nicotine Metabolite Ratio

In order to determine nicotine, cotinine and 3′-hydroxycotinine concentrations we analyzed salivary samples employing a common protocol [9,27]. After rinsing their mouths and sucking a lemon candy (Smint^®^) to stimulate saliva production, participants provided 9 mL of saliva by directly spitting in a test tube with the help of a funnel. Each individual sample was separated into 3 mL aliquots and stored at −20 °C. The frozen samples were sent to the Group of Integrative Pharmacology and Systems Neuroscience of the Municipal Institute for Medical Research (IMIM-Hospital del Mar) in Barcelona. All biomarkers were determined by alkaline single liquid-liquid extraction with dichloromethane/isopropanol followed by liquid chromatography-tandem mass spectrometry; this methodology is described elsewhere [28]. The limit of quantification of this method was 0.5 ng/mL for nicotine, 0.1 ng/mL for cotinine and 0.04 ng/mL for trans-3′-hydroxycotinine. Values under the limit of quantification were halved to avoid overestimation or underestimation bias. Then, the rate of nicotine metabolism, or RNM (cotinine/nicotine) and the nicotine metabolite ratio, or NMR (trans-3′-hydroxycotinine/cotinine) were calculated.

### 2.2. Smoking Status and Use of E-Cigarettes

According to self-reported information, we classified the participants into the five following groups: (a) dual users (participants who were both current cigarette smokers and users of e-cigarettes), (b) cigarette smokers, (c) e-cigarette exclusive users with nicotine, (d) e-cigarette exclusive users without nicotine, and (e) nonusers. The inclusion of users of e-cigarettes without nicotine and nonusers of any products is due to the fact that they can have low levels of nicotine metabolites, and can generally be attributable to passive exposure to nicotine [29]. Inclusion of non-users is justified since exposure to tobacco smoke was not controlled for.

Nonusers were individuals who declared to have never smoked or to have formerly smoked/used tobacco/nicotine products or e-cigarettes. Subjects were considered current cigarette smokers if they declared smoking cigarettes daily or occasionally (people who smoked regularly within a week but did not smoke every day of the week) at the moment of the survey. Any person who used e-cigarettes and did not smoke for at least six months was considered an exclusive e-cigarette user. If exclusive users of e-cigarettes declared that the e-liquid contained nicotine, regardless of the concentration, the users were categorized as “e-cigarette exclusive users with nicotine”. Otherwise, they were categorized into “e-cigarette exclusive users without nicotine”. Individuals who reported smoking cigarettes and using e-cigarettes were considered dual users.

### 2.3. Biological Variables

Our study also included information on self-reported biological variables, namely sex, age and body mass index (BMI). We categorized the individuals’ age into 3 groups (according to the sample tertiles): (a) between 18 and 44 years old, (b) between 45 and 64 years old and (c) between 65 and 89 years old. Similarly, we also categorized the individuals’ BMI in a total of 4 groups (in accordance to WHO guidelines [30], although the underweight range was extended to increase the sample size): (a) between 10 and 20 kg/m^2^, (b) between 21 and 25 kg/m^2^, (c) between 26 and 30 kg/m^2^ and (d) between 31 and 60 kg/m^2^.

### 2.4. Statistical Analysis

Because of the skewness in nicotine metabolites values [25], we calculated the geometric mean (GM) and geometric standard deviation (GSD) of RNM and NMR. Analyses were stratified by the five groups of smoking status and use of e-cigarette and biological variables. We compared both RNM and NMR across all groups using the Mann Whitney test for independent samples and Kruskal-Wallis H test, both with Bonferroni correction (multiplying the *p*-values by the number of comparisons) according to smoking status and use of e-cigarette, sex, age and BMI. Given the potential confounding effect of the daily number of cigarettes smoked and the variable for the e-cigarette, we realized two (adjusted and unadjusted) log-linear models for each one of the ratios according to the smoking and e-cigarette categories. We performed a sensitivity analysis to find differences between the complete sample and those participants with a cotinine level greater than the limit of quantification. To ease the interpretation of our results, we decided to follow Siegel et al. [20] methodology and classify participants based on overall RNM and NMR quartiles, labeling those in the first quartile as “slow metabolizers,” those in the second quartile as “moderate metabolizers,” and those in the third and fourth quartiles as “fast metabolizers.” The level of significance was set at α = 0.05. Data were analyzed using R (R Foundation for Statistical Computing, Vienna, Austria) version 4.0.4.

## 3. Results

The GM of RNM and NMR overall and stratified by smoking and e-cigarette use, sex, age and BMI are shown in Table 1. E-cigarette exclusive users without nicotine showed the lowest RNM value (GM: 0.08, *p*-values < 0.001). Cigarette smokers showed the highest ratio of them all (GM:2.08, *p*-values < 0.001). Nonusers have significantly higher values (GM:0.27, *p*-values < 0.001) than e-cigarette exclusive users without nicotine, but lower than dual users and e-cigarette exclusive users with nicotine. There was no significant difference between e-cigarette exclusive users with nicotine and dual users (GM:0.49 vs. GM:0.48). Regarding NMR, significant differences were found between cigarette smokers and nonusers (GM:0.27 vs. GM:0.23, *p*-values < 0.05).

Younger individuals showed higher RNM than older individuals (0.53 vs. 0.31). Similarly, individuals with BMI 21–25 have higher RNM than those in the higher categories (0.52 vs. 0.35–0.36). On the other hand, NMR was higher in females (0.24 vs. 0.21; *p*-value <0.001) and lower in younger individuals (0.21 vs. 0.25; *p*-value < 0.001). No differences in NMR were found between individuals according to BMI. The comparison of the unadjusted and the adjusted log-linear models for both the RNM and the NMR (Table 2) did not show much difference from each other, indicating that there is no significant confusion effect in any of the ratios. For the RNM, all the categories of smoking status and use of e-cigarette were significant (<0.01) in both models, while for the NMR only the category cigarette smokers (<0.01) was significant. The quartile division of the RNM classifies those individuals whose RNM is lower than 0.20 as slow metabolizers (first quartile), between 0.2 and 0.36 (second quartile) as moderate metabolizers and those with a value higher than 0.36 as fast metabolizers. In the case particular case of the NMR, the first quartile was 0.14 and the second 0.23.

## 4. Discussion

By describing the relationship between NMR and biological differences, previous studies have categorized individuals according to the rate of metabolism with which they metabolize nicotine and its derivatives [17,20]. The quartile division method of NMR for the classification of slow (<0.14), moderate (0.14–0.23) and fast metabolizers (>0.23) in saliva was in line with previous studies, on which the first and second quartile for NMR dividing between slow and moderate metabolizers were found to be 0.17 and 0.29 [31], and 0.18 and 0.3 [32], respectively. In contrast with slower metabolizers, faster metabolizers of nicotine are thought to clear nicotine much faster, develop more symptoms of nicotine dependence, and increase their nicotine dose in order to minimize withdrawal symptoms [20]. To the best of our knowledge, this is the only study comparing salivary nicotine rate of metabolism in Spain by tobacco consumption including e-cigarettes in the general population and also the first time that a division point in the RNM has been described with the intention of dividing a sample into different types of metabolizers. Regarding biological factors, previous studies showed that NMR is higher in females [33,34] and that females are faster metabolizers than males [7,13,33], while age [34] and body mass index (BMI) have been negatively associated with NMR [31]. In the particular case of the NMR, faster nicotine metabolism among nonusers as compared to cigarette smokers has been reported [33].

### 4.1. Nicotine Metabolite Ratio & Rate of Nicotine Metabolism According to Smoking Status and Use of E-Cigarette

Regarding the NMR and according to our results, smokers, users of e-cigarettes (with and without nicotine) and nonusers in our sample are moderate metabolizers, while dual users are fast metabolizers, having a higher NMR, which is the opposite of expectations, since the NMR should be lower in consumers of tobacco products [35]. Our findings when comparing the adjusted and unadjusted linear models indicate no confounding effect attributable to the number of cigarettes per day. Consistent with the findings on NMR, it is possible that the discrepancies observed in dual users and users of e-cigarettes may be attributable to variations in tobacco consumption topography [32,36]. In this sense, future studies must investigate the relationship between nicotine metabolization ratios with the consumption topography of different tobacco products.

On the other hand, regarding the RNM, results show that e-cigarette (without nicotine) users are slow metabolizers, nonusers are moderate metabolizers, and smokers, e-cigarette (with nicotine) users and dual users are fast metabolizers. Given the higher nicotine dependence of fast metabolizers [20], our results support the previously described hypothesis of faster metabolizers taking a higher nicotine dose in order to alleviate withdrawal symptoms. Due to the low nicotine concentration in nonusers and users of e-cigarettes without nicotine, perhaps there are differences in the nicotine metabolization between passive smokers depending on the level of exposure. However, the GM of RNM for users of e-cigarettes without nicotine is extremely low. Results for this unstable rate may not be representative of the whole population.

In line with a previous study [7], NMR showed utility as a biomarker of nicotine metabolism in users of e-cigarettes. In this sense, it is important to continue studying the similitudes in the nicotine metabolism between users of e-cigarettes and cigarette smokers through NMR and RNM in the future.

### 4.2. Nicotine Metabolite Ratio & RNM According to Biological Factors

We found significant differences in nicotine metabolism according to sex in NMR (*p*-values < 0.001); the quartile division in our sample suggests that female are fast metabolizers and male are moderate metabolizers. However, no differences were found when comparing the GM of RNM between males and females. Another study suggests that females are more likely to have faster nicotine metabolism and have higher dependence on nicotine products, possibly due to the effect of sex hormones on CYP2A3 [12,13]. Nevertheless, the GM of NMR for female (0.24) and male (0.21) obtained in our study is lower than the one reported in that study (0.43 and 0.35, respectively) [13]. Discrepancies between both studies may be due to the differences between the two populations, as their sample was composed of failed quitters and ours of cigarette smokers, users of e-cigarettes, and nonusers, and, as previously reported, when the NMR is higher, nicotine metabolization is faster, and faster metabolizers may have a much harder time quitting smoking [37].

When comparing the GM of RNM by age, a negative association was clearly observed, being older individuals (65–89 years) faster nicotine metabolizers than younger ones (<64 years). NMR showed a positive association with age, but just between participants <44 years (moderate metabolizers) and participants 65–89 years (fast metabolizers) individuals. A previous study reported a nicotine clearance similar to the one we observed in RNM [38]. However, that study counted with limited subjects (*n* = 40) and was conducted introducing nicotine via intravenous infusion and measured in plasma and urine, so differences in nicotine metabolization between different biological matrixes are to be expected. As previous studies reported similar trends concerning age and rates of nicotine metabolism, our results suggest that accumulated exposure to nicotine and natural metabolic changes associated with age may enhance its metabolization rate [12,13]. In this case, the differences observed are negatively associated with age, being the value for older individuals (65–89 years) practically the same as nonusers.

Our results also shows that there is a negative association between the RNM and the BMI, while no association was found between the NMR and the BMI, although the quartile analysis categorized overweight and underweight individuals as fast metabolizers (>0.23), while the rest were categorized as moderate metabolizers. Previous studies with a similar sample size reported BMI being negatively associated with NMR [14,31,39]. However, understanding the effect and metabolic rates of novel forms of nicotine intake in smoking status, use of e-cigarette and biological factors needs further investigation.

Discrepancies between both ratios could be attributed to NMR with independence of the number of cigarettes smoked [17]. Based on this, we should use the most stable ratio (NMR) as a good unbiased indicator of nicotine metabolization. However, if reported together, they could be used to determine the differences and have a rough estimation of smoking quantities.

### 4.3. Limitations

Our study has some limitations that should be mentioned. Although the questionnaire has been self-declared and could represent a reporting bias, there is sufficient evidence that self-reported data on smoking performs well when working with trained interviewers. In addition, as one portion of the pooled data was taken from a longitudinal study (this sample is aged) and the other portion used a non-probabilistic sampling technique, our sample is not representative of the general population. We did not analyze nicotine concentration in the e-cigarettes to compare with the self-reported data. Neither did we control for passive exposure. Furthermore, classifying individuals in the sample by quartiles has the downside that the selection of the sample influences that classification. Also, there is female underrepresentation in our sample, and as females are faster metabolizers than males, our results may present underestimation bias. Lastly, there are some significant concerns about the use of metabolite ratios that must be mentioned. One of them is that the NMR may not be reliable when calculated using values that are below the limit of quantification, not only because small measurement errors can lead to large differences in ratios but also because such low values indicate that cotinine and trans-3′-hydroxycotinine are not at steady state in the body. When not at steady state, the concentrations of trans-3′-hydroxycotinine are not solely formation-dependent and therefore the assumptions underlying the use of the NMR as a measure of nicotine metabolism are not met [40]. However, we performed a sensitivity analysis between the complete sample size and those participants with a cotinine level greater than the limit of quantification and could not find significant differences between groups. Other potential issue related to the use of metabolite ratios is that the RNM is highly dependent on time since last use which is why it is not commonly used as a measure of nicotine metabolism rate. Regrettably, time since last use is not found among the data obtained through the questionnaires passed.

## 5. Conclusions

We did not find significant differences in the NMR between dual users, e-cig (with nicotine) users, and cigarette smokers or between nonusers and e-cigarette (without nicotine) users; however, we successfully identified different types of metabolizers according to smoking status and use of e-cigarettes. Both in RNM and NMR, dual users were fast metabolizers. At the level of absorption and metabolic rates, the use of e-cigarettes with nicotine could be analogous to the use of conventional tobacco products. These findings warrant further investigation given the potential of the NMR to inform about the biotransformation of nicotine and, consequently, about smoking status and biological differences in nicotine metabolic rate between individuals.

## Figures and Tables

**Table 1:** Geometric mean (GM) and Geometric Standard Deviation (GSD) of Rate of Nicotine Metabolism (RNM) and Nicotine Metabolite Ratio (NMR) in saliva samples according to smoking status and use of e-cigarette, sex, age and body mass index (BMI).

	*n* (%)	RNM GM (GSD)	NMR GM (GSD)
Overall	974	0.43 (4.27)	0.22 (2.09)
Smoking and e-cigarette status ^a^			
Nonusers of any product ^a5^	508 (52.16)	0.27 (2.30) ^(a1, a2, a3, a4)^ ***	0.23 (1.99) ^a2^ *
E-cigarette exclusive users without nicotine ^a4^	41 (4.21)	0.08 (8.12) ^(a1, a2, a3, a5)^ ***	0.23 (1.80)
E-cigarette exclusive users with nicotine ^a3^	164 (16.84)	0.49 (3.45) ^(a2, a4, a5)^ ***	0.22 (1.90)
Dual users ^a1^	95 (9.75)	0.48 (4.70) ^(a2, a4, a5)^ ***	0.24 (1.80)
Cigarette smokers ^a2^	166 (17.04)	2.08 (4.90) ^(a1, a3, a4, a5)^ ***	0.18 (2.61) ^a5^ *
Sex ^b^			
Female ^b1^	442 (45.38)	0.43 (3.98)	0.24 (2.11) ^b2^ ***
Male ^b2^	532 (54.62)	0.43 (4.52)	0.21 (2.06) ^b1^ ***
Age (years) ^c^			
18–44 ^c1^	371 (38.09)	0.53 (4.97) ^c3^ ***	0.21 (1.96) ^c3^ ***
45–64 ^c2^	363 (37.27)	0.42 (4.17) ^c3^ **	0.22 (2.13)
65–89 ^c3^	240 (24.64)	0.31 (3.18) ^c1^ ***^, c2^ **	0.25 (2.20) ^c1^ ***
BMI (kg/m^2^) ^d^			
10–20 ^d1^	64 (6.57)	0.54 (4.93)	0.26 (2.07)
21–25 ^d2^	378 (38.81)	0.52 (4.56) ^d3^ ***^, d4^ **	0.22 (2.12)
26–30 ^d3^	336 (34.50)	0.35 (3.93) ^d2^ ***	0.22 (2.09)
31–60 ^d4^	186 (49.21)	0.36 (3.86) ^d2^ **	0.23 (2.01)

Letters as superscripts (^a^, ^b^, ^c^, and ^d^) indicate the qualitative variable (Smoking and e-cigarette status, Sex, Age (years), and BMI (kg/m^2^) respectively). Numbers as superscripts indicate the level of the variable whose letter precedes them. The superscripts for statistical significance (* significant at *p* < 0.050; ** significant at *p* < 0.010; *** significant at *p* < 0.001) report on the significant *p*-values (after adjusting by Bonferroni correction) when comparing the categories within each variable to which that row corresponds with the rest of the categories within that same variable, always within the same category.

**Table 2:** Log-linear models for Smoking and e-cigarette status (nonusers as reference) for Rate of Nicotine Metabolism (RNM) and Nicotine Metabolite Ratio (NMR). The first one (Unadjusted Model) and the second one with its presence (Adjusted Model).

Title 1	RNM			NMR		
Log-linear Models	exp(Estimate) ^c^	CI ^d^	*p*-Value	exp(Estimate) ^c^	CI ^d^	*p*-Value
Unadjusted model ^a^						
Intercept	0.27	0.24; 0.30	<0.001	0.23	0.22; 0.25	<0.001
E-cigarette exclusive users without nicotine	0.08	0.05; 0.14	<0.001	0.23	0.17; 0.32	0.96
E-cigarette exclusive users with nicotine	0.49	0.36; 0.68	<0.001	0.22	0.18; 0.27	0.37
Dual users	0.48	0.33; 0.70	<0.001	0.24	0.19; 0.30	0.71
Cigarette smokers only	2.08	1.51; 2.85	<0.001	0.18	0.15; 0.22	<0.001
Adjusted model ^b^	0.27	0.24; 0.30	<0.001	0.23	0.22; 0.25	<0.001
Users of e-cigarettes without nicotine	0.09	0.05; 0.14	<0.001	0.23	0.17; 0.32	0.97
Users of e-cigarettes with nicotine	0.65	0.45; 0.95	<0.001	0.21	0.16; 0.26	0.18
Dual	0.60	0.4; 0.89	<0.001	0.23	0.18; 0.29	0.91
Cigarette smokers	2.43	1.74; 3.39	<0.001	0.18	0.14; 0.22	<0.001
Daily number of cigarettes smoked	0.26	0.23; 0.29	<0.001	0.23	0.22; 0.25	0.29

^a^ Log-linear model computed without including the daily number of cigarettes as covariable. ^b^ Log-linear model adjusted for the daily number of cigarettes and its equivalent for e-cigarette. ^c^ Exponential of the sum of the estimates (but for the daily number of cigarettes). ^d^ Confidence Interval.

## Data Availability

The data that support the findings of this study are available on request from the corresponding author.
